# Biliary drainage improves the predictive value of modified Glasgow Prognostic Scores in inoperable pancreatic cancer

**DOI:** 10.1371/journal.pone.0178777

**Published:** 2017-06-23

**Authors:** Chikara Iino, Tadashi Shimoyama, Takasato Igarashi, Tomoyuki Aihara, Kentaro Ishii, Juichi Sakamoto, Hiroshi Tono, Shinsaku Fukuda

**Affiliations:** 1 Department of Internal medicine, Hirosaki Municipal Hospital, Hirosaki, Japan; 2 Department of Gastroenterology, Hirosaki University Graduate School of Medicine, Hirosaki, Japan; Virginia Mason Medical Center, UNITED STATES

## Abstract

**Objective:**

To assess the influence of biliary drainage to cholangitis on modified Glasgow Prognostic Score (mGPS) in patients with pancreatic cancer.

**Methods:**

mGPS was calculated before and after biliary drainage in 47 consecutive patients with inoperable pancreatic cancer who were receiving chemotherapy. Biliary drainage was indicated for malignant obstructive jaundice that prevented the administration of chemotherapy. To elucidate mGPS values, serum levels of CRP and albumin were measured at the time of diagnosis (before biliary drainage). Overall survival was evaluated and risk factors, which contribute to overall survival, were examined.

**Results:**

Biliary drainage was performed in 15 patients. Using values obtained before biliary drainage, there were no significant differences in median survival time between patients with a mGPS of 0 and those with a mGPS of 1 or 2 (10.7 vs. 9.4 months; p = 0.757). However, using values obtained after biliary drainage, median survival time was significantly higher in patients with a mGPS of 0 than in those with a mGPS of 1 or 2 (11.4 vs. 4.7 months; p = 0.002). Multivariate analysis revealed that a mGPS of 1 or 2 (HR: 3.38; 95% CI: 1.35–8.46, p = 0.009), a carbohydrate antigen 19–9 >1000 U/mL (2.52; 1.22–5.23, p = 0.013), a performance status of 2 (7.68; 2.72–21.28, p = 0.001), carcinoembryonic antigen level >10 ng/mL (2.29; 1.13–4.61, p = 0.021) were independently associated with overall survival.

**Conclusion:**

mGPS values obtained after biliary drainage appear to be a more reliable indicator of overall survival in patients with inoperable pancreatic cancer.

## Introduction

It is widely recognized that cancer patient outcome is not solely dependent on tumor-related factors, but several patient-related factors play an important role. The Glasgow Prognostic Score (GPS) is arrived at by analysis of circulating levels of C-reactive protein (CRP) and serum albumin, and is a useful indicator of prognosis in various malignancies [1]. Moreover, recent reports have demonstrated that a modified version of the GPS (termed modified GPS, or mGPS) is superior to the GPS [2–5]. However, few studies have evaluated the usefulness of the mGPS as a prognostic tool in patients with inoperable pancreatic cancer. At present, use of the mGPS is not internationally accepted, and its usefulness in patients with pancreatic cancer remains controversial.

In some patients with pancreatic cancer, biliary obstruction leads to the development of cholangitis. In these patients, enhanced inflammatory responses may influence mGPS values. However, studies that have examined the usefulness of mGPS values in inoperable pancreatic cancers have done so at the time of diagnosis, and thus, before biliary drainage. The aim of the present study was to elucidate whether, in patients with inoperable pancreatic cancer, the prognostic value of mGPS values are altered following biliary drainage for cholangitis.

## Materials and methods

We conducted a retrospective study of 47 consecutive patients receiving chemotherapy of Gemcitabine or S-1 (tegafur, gimeracil, and potassium oxonate) for inoperable pancreatic cancer at Hirosaki Municipal Hospital between August 2010 and February 2015. The characteristics of the subjects are shown in Table 1. This group had a median age of 75.8 (66–80) years, 24 were male, and 28 had metastatic lesions. Biliary drainage was indicated for malignant obstructive jaundice that prevented the administration of chemotherapy. Biliary drainage was performed using self-expandable metal or tube stents.

**Table 1:** Patients characteristics.

Age		75.8 (66–80)
Sex	Male/Female	24/23
Performance status	0/1/2	24/14/9
Location	Head/Body/Tail	28/13/6
Reason for inoperable (%)	Locally invasion	19 (40.4%)
	Metastatic	28 (59.6%)
End-point	Alive/Died	4/43
First line chemotherapy (%)	GEM	27 (57.4%)
	GEM+S-1	16 (34.1%)
	S-1	4 (8.5%)
CEA (ng/mL)		9.6 (4.9–26.6)
CA19-9 (U/mL)		618 (81.5–7233)
Hb (g/dL)		12.5 (11.2–13.4)
T-bil (mg/dL)		1 (0.6–6.9)

Quantitative variables are shown as median (interquartile range) GEM: gemcitabine, EBD: endoscopic biliary drainage, PBD: percutaneous transhepatic biliary drainage, CEA: carcinoembryonic antigen, CA19-9: carbohydrate antigen 19–9, Hb: hemoglobin, T-Bil: total bilirubin, mGPS: modified Glasgow Prognostic Score, CI: confidence interval

To elucidate mGPS values, serum levels of CRP and albumin were measured at the time of diagnosis (before biliary drainage) and when total serum bilirubin levels had decreased to less than 3 mg/dL (measured in biliary drainage). Patients who showed both elevated CRP levels (>1.0 mg/dL) and hypoalbuminemia (<3.5 g/dL) were allocated a score of 2, patients with an elevated CRP level only (>1.0 mg/dL) were allocated a score of 1, and patients with neither of these factors were allocated a score of 0 [2].

Overall survival was compared between the patients with a mGPS of 0 and those with a mGPS of 1 or 2 before and after biliary drainage. Moreover, overall survival was also compared in the 32 patients without jaundice.

Statistical analysis was performed using the Statistical Package for the Social Science (SPSS) version 20.0 (SPSS Inc., Chicago, IL, USA). Overall survival rates were calculated from the date of diagnosis to death from cancer. Date censor date was 1st February 2016. Survival curves were analyzed using the Kaplan-Meier method based on mGPS before and after biliary drainage, and the differences were compared using a log-rank test. Cox regression analysis was used to identify factors which associated with overall survival. The significant variables in univariate analysis were selected for further evaluation using the multivariable Cox proportional hazard model. Results were considered statistically significance when p < 0.05.

The study was approved by ethics committee of Hirosaki Municipal Hospital.

## Results

**Fig 1:**
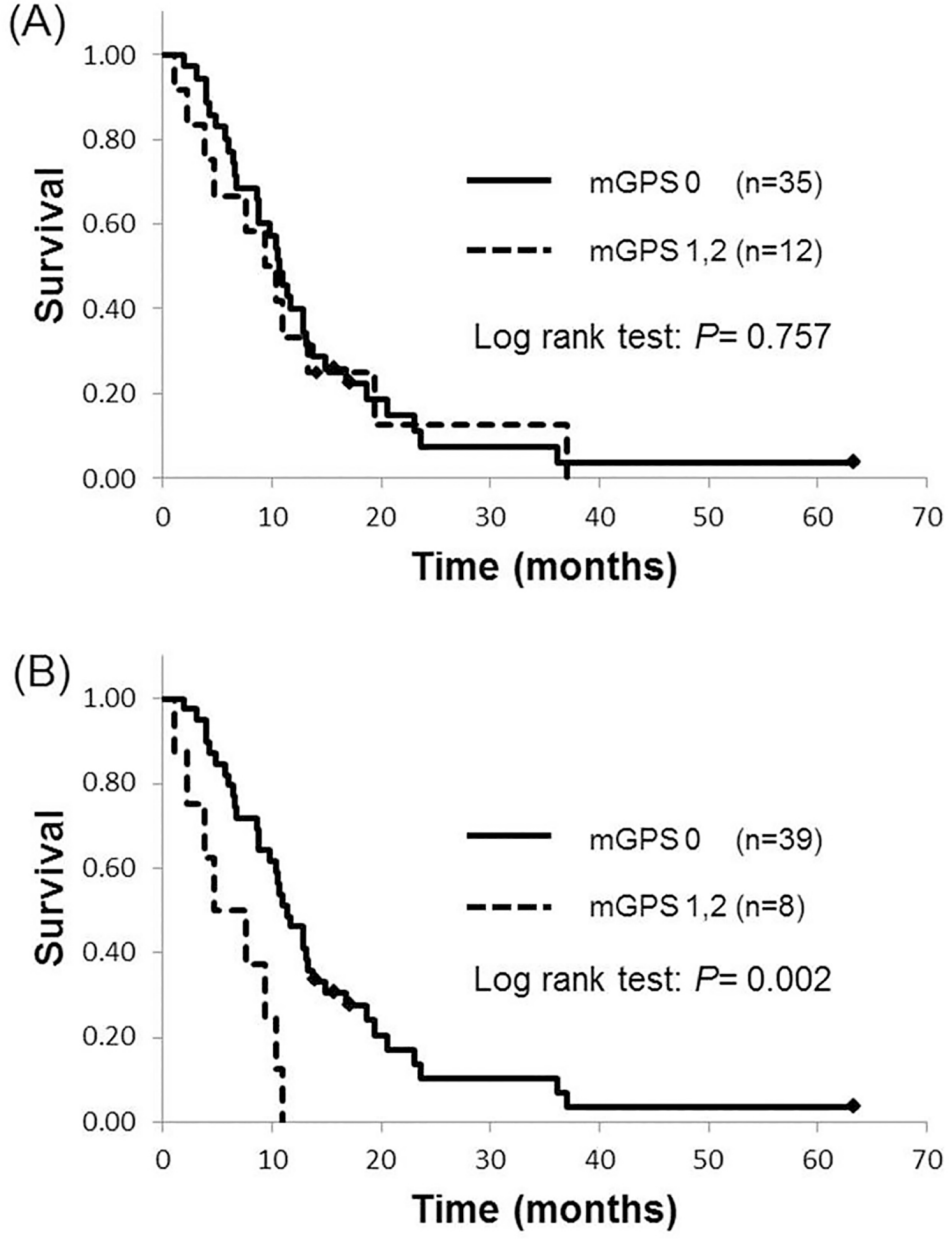
Kaplan-Meier curves for overall survival by mGPS values obtained prior to biliary drainage A) and after biliary drainage B).

**Fig 2:**
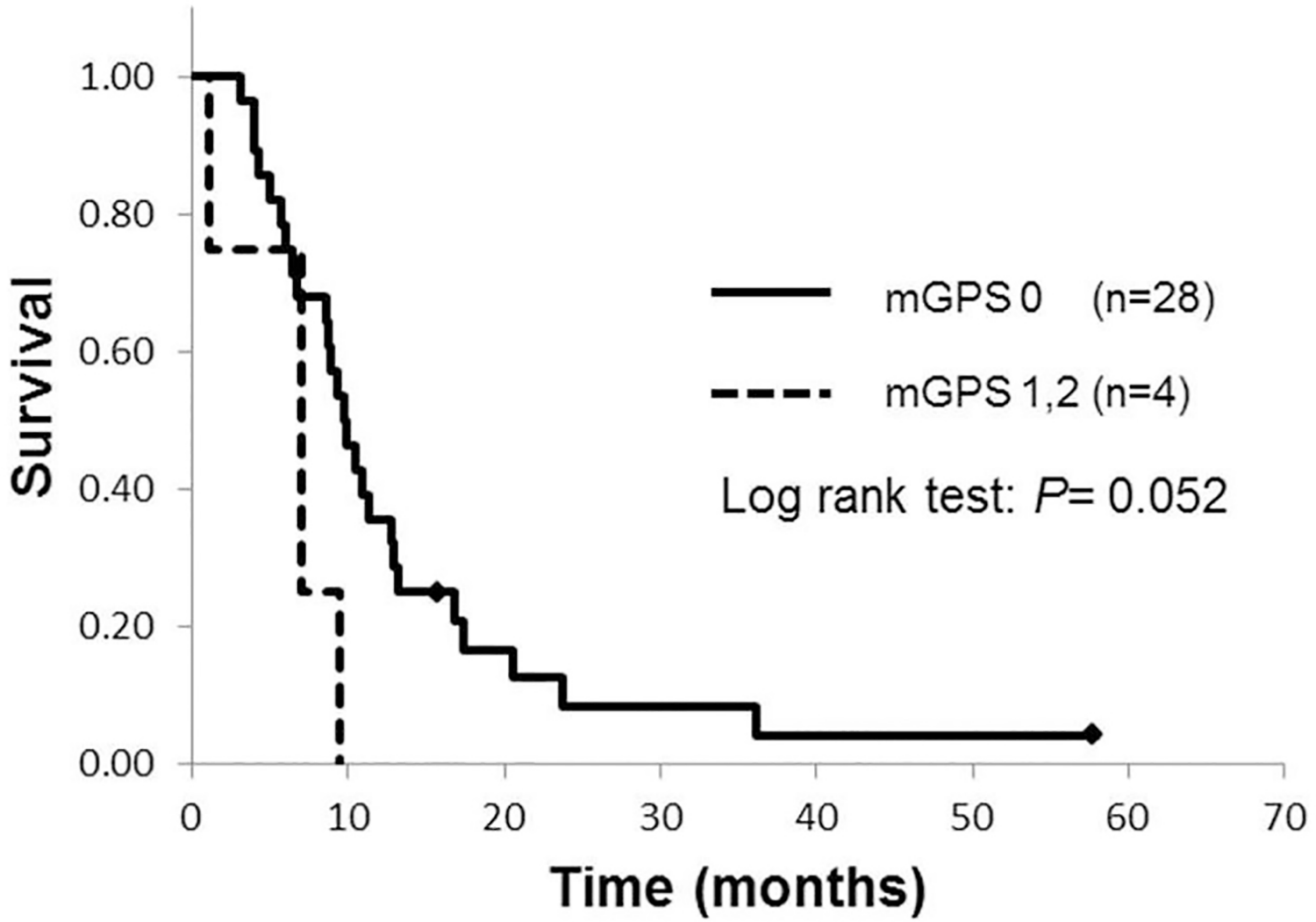
Kaplan-Meier curves for overall survival by mGPS values without obstructive jaundice.

At the time of data analysis, 43 of the initial patients had died. The median survival time (MST) for all patients was 10.5 months and 18 patients were alive at 12 months. Biliary drainage was performed in 15 patients. Fourteen patients received endoscopic transpapillary biliary drainage (EBD), and one patient underwent percutaneous transhepatic biliary drainage (PTD) as a result of duodenal invasion. At the time of the diagnosis, and before drainage, a mGPS of 0 was observed in 35 patients, a mGPS of 1 was observed in 5 patients and a mGPS of 2 was observed in 7 patients. Kaplan-Meier analysis revealed no significant difference in MST between patients with a pre-drainage mGPS of 0 and those with a pre-drainage mGPS 1 or 2 (10.7 months vs. 9.4 months; p = 0.757) (Fig 1A). After biliary drainage in 15 patients, the mGPS decreased from 1 to 0 in 2 patients and the mGPS decreased from 2 to 0 in 2 patients. Therefore, a mGPS of 0 was observed in 39 patients, a mGPS of 1 in 3 patients and a mGPS of 2 in 5 patients. After biliary drainage, patients with a mGPS of 0 demonstrated significantly greater survival (MST: 11.4 months) compared to those with a mGPS of 1 or 2 (MST: 4.7 months) (p = 0.002) (Fig 1B). However, in the analysis of 32 patients without jaundice, Kaplan-Meier analysis revealed no significant difference in MST between patients with a mGPS of 0 and those with a mGPS 1 or 2 (9.7 months vs. 6.9 months; p = 0.052) (Fig 2).

In univariate analysis, performance status (PS), extent of disease, serum levels of carcinoembryonic antigen (CEA), serum levels of cancer antigen 19–9 (CA19-9), and mGPS values after biliary drainage were significantly associated with overall survival (Table 2). Results of multivariate analysis using these variables are shown in Table 3. A mGPS of 1 or 2 (HR: 3.38; 95% CI: 1.35–8.46, p = 0.009), a PS of 2 (HR: 7.68; 95%CI: 2.72–21.28, p = 0.001), CA19-9 > 1,000 U/mL (HR: 2.52; 95% CI: 1.22–5.23, p = 0.013) and CEA > 10 ng/mL (HR: 2.29; 95% CI: 1.13–4.61, p = 0.021) were all independently associated with overall survival.

**Table 2:** Univariate analysis in relation to overall survival.

Variables	*p* value	Hazard ratio	95% CI
Age(years) ≧75	0.460	1.27	0.68–2.38
Sex (male / female)	0.274	1.42	0.76–2.66
Performance status = 2	<0.001	7.95	3.41–18.53
Location(head / body or tail)	0.347	0.74	0.39–1.39
Extent of disease (Locally advanced / Metastasis)	0.020	2.11	1.13–3.97
Chemotherapy (GEM or GEM+S-1 / S-1)	0.854	1.12	0.34–3.65
Hb <11 g/dl	0.372	0.71	0.34–1.50
T-Bil >2.0 mg/dl	0.538	0.82	0.44–1.54
CEA > 10 ng/ml	0.018	2.12	1.14–3.95
CA19-9 > 1,000 U/ml	0.003	2.65	1.40–5.02
mGPS = 1 or 2 before biliary drainage	0.756	1.12	0.56–2.22
mGPS = 1 or 2 after biliary drainage	0.002	3.71	1.60–8.60

GEM gemcitabine, Hb hemoglobin, T-Bil total bilirubin, CEA carcinoembryonic antigen, CA19-9 carbohydrate antigen 19–9, mGPS modified Glasgow Prognostic Score, 95% CI 95% confidence interval

**Table 3:** Multivariate analysis in relation to overall survival.

Variables	*p* value	Hazard ratio	95% CI
performance status = 2	0.001	7.68	2.72–21.28
CEA >10 ng/mL	0.021	2.29	1.13–4.61
CA19-9 > 1,000 U/mL	0.013	2.52	1.22–5.23
mGPS = 1 or 2 after biliary drainage	0.009	3.38	1.35–8.46

CEA carcinoembryonic antigen, CA19-9 carbohydrate antigen 19–9, mGPS modified Glasgow Prognostic Score, 95% CI 95% confidence interval

## Discussion

In patients with inoperable pancreatic cancer, biliary drainage appears to improve the prognostic value of mGPS scores. Patients with a mGPS of 0 after biliary drainage had greater survival compared to those with GPS 1 or 2. A mGPS of 1 or 2—even after biliary drainage—was associated with a poor prognosis. To date, only four retrospective studies have evaluated the usefulness of the GPS or mGPS in patients with inoperable pancreatic cancer [6–9]. In these studies, mGPS was assessed at the time of pancreatic cancer diagnosis and thus before performing biliary drainage. The present study showed that calculation of the mGPS after biliary drainage provides more accurate prognostic data compared to mGPS obtained prior to biliary drainage. The improvement results from the biliary drainage for accompanied cholangitis. Of the 15 patients who underwent biliary drainage, the mGPS values decreased in 4 cases in which patients survived beyond 9 months (average overall survival was 19.6 months). Therefore, the higher mGPS scores obtained prior to biliary drainage may misrepresent the prognosis of the patients. Thus, patients who have accompanying cholangitis should be evaluated using mGPS values obtained after biliary drainage. We also assessed the utility of the mGPS in patients without jaundice but overall survival was not significantly different between the patients with a mGPS of 0 and those with a mGPS 1 or 2. However, the patients with a mGPS 1 or 2 tended to have poor prognosis. There were only 4 patients with a mGPS 1 or 2. Small sample size might preclude us to find significant difference in patients without jaundice.

In our multivariate analysis, a poor prognosis was associated with a mGPS of 1 or 2, as well as a PS of 2, serum CEA >10 ng/mL, and serum CA19-9 >1,000 U/mL. A mGPS of 1 or 2 resulted in higher hazard ratio than tumor makers. In addition, serum measurement of both CRP and albumin is less expensive than tumor markers. Therefore, mGPS may be valuable as a tumor marker in predicting the prognosis of patients with inoperable pancreatic cancer, on the provision that mGPS is calculated after biliary drainage.

Our study has several limitations. First, this is retrospective analysis of a small number of patients in a single institution. Indeed, only 15 of the 47 patients underwent biliary drainage. Therefore, although our results suggested the biliary drainage appears to improve the prognostic value of mGPS scores, further studies with large number of patients are required to validate the results of this study. Second, 3 chemotherapy regimens were used; 27 patients received gemcitabine, 16 patients received gemcitabine plus S-1, and 4 patients received S-1. A previous study suggested the non-inferiority of S-1 to gemcitabine and the non-superiority of gemcitabine plus S-1 [10]. Indeed, in our univariate analysis, the use of S-1 alone was not significantly associated with overall survival (*p* = 0.854).

## Conclusions

The prognostic value of mGPS is enhanced after biliary drainage in patients with inoperable pancreatic cancer. A mGPS of 1 or 2 after biliary drainage would suggest poor prognosis.

## Supporting information

S1 Table The data for study subjects. (XLSX)

S2 Table The data for multivariate analysis. (XLSX)

S1 Fig The data for Kaplan-Meier curves before and after biliary drainage. (XLSX)

S2 Fig The data for Kaplan-Meier curves without obstructive jaundice. (XLSX)
